# Long non-coding RNA DSCR8 acts as a molecular sponge for miR-485-5p to activate Wnt/β-catenin signal pathway in hepatocellular carcinoma

**DOI:** 10.1038/s41419-018-0937-7

**Published:** 2018-08-28

**Authors:** Yufeng Wang, Liankang Sun, Liang Wang, Zhikui Liu, Qing Li, Bowen Yao, Cong Wang, Tianxiang Chen, Kangsheng Tu, Qingguang Liu

**Affiliations:** Department of Hepatobiliary Surgery, The First Affiliated Hospital of Xi’an Jiaotong University, 710061 Xi’an, Shaanxi Province China

## Abstract

Previous evidences reveal that long non-coding RNA (lncRNA) down syndrome critical region 8 (DSCR8) involves in the progression of multiple cancers. However, the exact expression, function, and mechanism of DSCR8 in hepatocellular carcinoma (HCC) remain uncovered. In this study, real-time PCR in HCC tissues and cell lines indicated that DSCR8 expression was upregulated, while miR-485-5p was downregulated. MTT assay, plate clone formation, Edu assay, flow cytometry, and in vivo experiments indicated that DSCR8 promoted HCC cell proliferation and cycle, whereas accelerated cell apoptosis. Luciferase reporter gene assay, RIP assay, and rescue experiments demonstrated that DSCR8 functioned as a competing endogenous RNA (ceRNA) by sponging miR-485-5p in HCC cells. Furthermore, gain- and loss-of-function studies showed that miR-485-5p activated Wnt/β-catenin signal pathway by targeting Frizzled-7 (FZD7). Moreover, DSCR8 activated Wnt/β-catenin signal pathway to promote HCC progression by DSCR8/miR-485-5p/FZD7 axis. Statistical analysis revealed that DSCR8 and miR-485-5p were closely related to some malignant clinicopathological features and 5-year survival rates of HCC patients. Taken together, the present study reports for the first time that DSCR8 activates Wnt/β-catenin signal pathway to promote HCC progression by DSCR8/miR-485-5p/FZD7 axis. The findings provide promising and valuable strategies for targeted therapy of HCC.

## Introduction

As one of the most common cancers in the world, hepatocellular carcinoma (HCC) has characteristics of high morbidity and high mortality^1–3^. In the past decades, though researchers have been long committed to identifying the potential therapeutic targets to improve the diagnosis and treatment levels for HCC, the outcomes of HCC patients remain unsatisfactory^2^. Thus, it is important for us to discover some novel and practical therapeutic targets for HCC.

In recent years, non-coding RNAs, including long non-coding RNAs (lncRNAs) and microRNA (miRNAs), have been largely reported in studies about cancers, including HCC^4,5^. In our previous studies, we found that some lncRNAs, such as CASC2^6^, TUSC7^7^, and Ftx^8^, act as competing endogenous RNAs (ceRNAs) to regulate HCC cells' migration, invasion, proliferation, apoptosis, and so on. For example, we found that lncRNA CASC2 exerts its inhibitory effects on HCC cells through CASC2/miR-367/FBXW7 pathway^6^. And we also found that lncRNA TUSC7 acts as a molecular sponge for miR-10a to suppress HCC cells' migration and invasion^7^. It is worth noting that, recently, lncRNA down syndrome critical region 8 (DSCR8) has been found to be dysregulated in uterine cancer and melanoma^9,10^. In these cancers, DSCR8 is highly expressed and might be potential prognostic indicators and therapeutic targets^9,10^. However, the expression and functions of DSCR8 in HCC remain unknown. MiR-485-5p has been identified as an anti-oncogene in HCC, which is involved in multiple biological and pathological processes of HCC^11,12^. However, the underlining mechanisms of miR-485-5p remain to be further explored.

Frizzled-7 (FZD7) is one of the receptors for Wnt signaling pathway^13^. It has been strongly confirmed that FZD7 is highly expressed in multiple cancers, including HCC^14–16^. And overexpressed FZD7 promotes the progression of cancers by inducing the activation of Wnt signaling pathway^13,17^. Recently, Wu J et al. found that miR-485-5p represses invasion and proliferation of melanoma cells by targeting FZD7^18^. However, whether FZD7 is regulated by miR-485-5p in HCC is still uncovered.

In the present study, we attempted to explore the expression, clinical significance, functions, and potential mechanisms of DSCR8 in HCC. DSCR8 was determined to be highly expressed in HCC. Gain- and loss-of-function analysis revealed that DSCR8 promoted cell proliferation and cell cycle, whereas suppressed cell apoptosis in HCC. Furthermore, the relationships among DSCR8, miR-485-5p, FZD7, and Wnt/β-catenin signal pathway in HCC cells were investigated. We found that DSCR8 activated Wnt/β-catenin signal pathway to promote HCC progression by DSCR8/miR-485-5p/FZD7 axis. DSCR8 and miR-485-5p were closely related to some malignant clinicopathological features and prognosis of HCC patients. In conclusion, DSCR8/miR-485-5p/FZD7 signal pathway may provide a novel and promising treatment strategy for HCC.

## Results

### Expression and clinical significance of DSCR8 in HCC

**Fig. 1:**
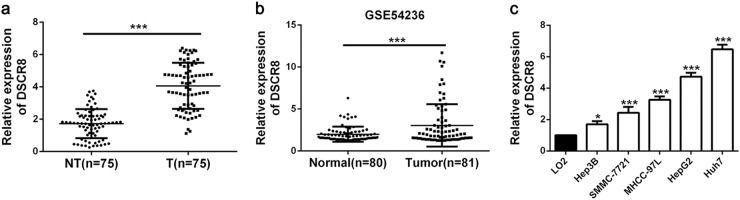
The expression of DSCR8 in HCC tissues and cell lines. **a** The real-time PCR results from our patients cohort revealed that DSCR8 was significantly upregulated in HCC tissues (T, *n* = 75) than that in normal tissues (NT, *n* = 75). **b** Data from GEO dataset (GSE54236) showed that the expression of DSCR8 in HCC tissues (*n* = 81) was significantly higher than that in normal tissues (*n* = 80). **c** DSCR8 expression was upregulated in HCC cell lines compared to normal hepatocyte (LO2). *P *<* 0.05, ***P < 0.001

The expression of DSCR8 in HCC tissues was detected by real-time PCR. And we found that the median expression of DSCR8 was much higher in HCC tissues than that in non-tumor tissues (*P* < 0.001, Fig. 1a). Then we analyzed the data from GEO dataset (GSE54236). Interestingly, the data displayed that DSCR8 was frequently upregulated in HCC tissues (*P* < 0.001, Fig. 1b), which was consistent with our finding. Additionally, DSCR8 was overexpressed in HCC cell lines compared to that in human normal liver cell (LO2) (*P* < 0.05, *P* < 0.001, respectively, Fig. 1c). Taken together, we conclude that DSCR8 might be an oncogene in HCC.

### DSCR8 promotes cell proliferation and cell cycle and inhibits cell apoptosis in HCC

**Fig. 2:**
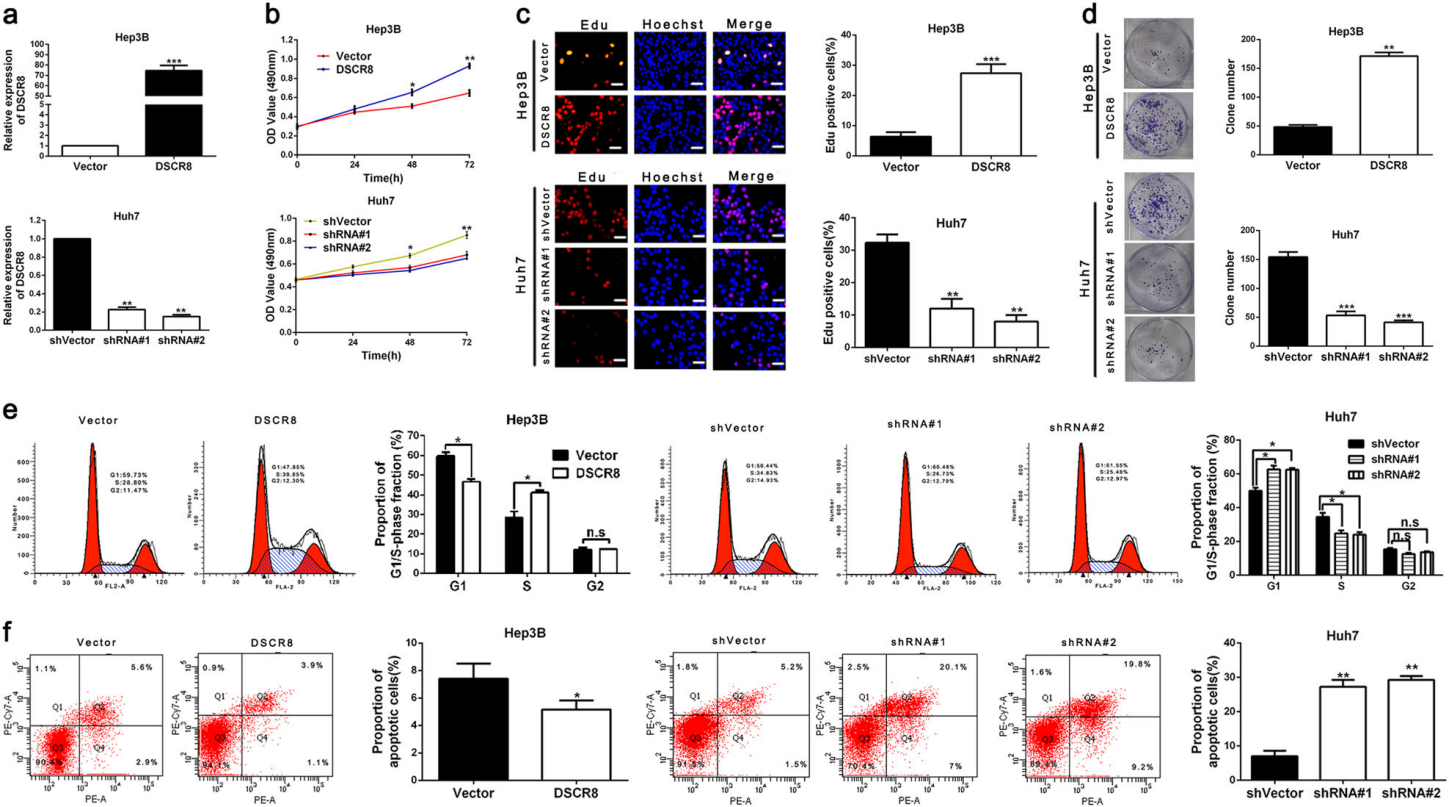
DSCR8 promotes HCC cells proliferation, cells cycle, and inhibits cells apoptosis in vitro. **a** DSCR8 expression of Hep3B cells was increased by pcDNA/DSCR8, and DSCR8 expression of Huh7 cells was decreased by DSCR8 shRNAs (shRNA#1, shRNA#2). pcDNA/DSCR8 accelerated, whereas DSCR8 shRNAs inhibited HCC cells proliferation (**b**, **c**), colony formation (**d**), and cell cycle (**e**). **f** pcDNA/DSCR8 repressed, whereas DSCR8 shRNAs induced HCC cells apoptosis. *n* = three independent experiments. Bars: 50 µM. *P < 0.05, **P < 0.01, ***P < 0.001

We increased the DSCR8 expression of Hep3B cells by pcDNA/DSCR8 and decreased the DSCR8 expression of Huh7 cells by sh-DSCR8. As shown in Fig. 2a, the transfection efficiencies were validated by real-time PCR. Then 3-[4,5-dimethylthiazol-2-yl]-2,5 diphenyl tetrazolium bromide (MTT) assay, 5-ethynyl-2¢-deoxyuridine (Edu) assay, plate clone formation assay, and flow cytometry for detection of cell cycle were conducted to assess the effects of DSCR8 on cell proliferation and cell cycle in HCC cells. Results showed that pcDNA/DSCR8 accelerated the proliferation (Fig. 2b–d) and cell cycle (Fig. 2e) of Hep3B cells, while sh-DSCR8 inhibited the proliferation (Fig. 2b–d) and cell cycle (Fig. 2e) of Huh7 cells. In addition, flow cytometry for detection of cell apoptosis revealed that pcDNA/DSCR8 repressed apoptosis of Huh7 cells (Fig. 2f), while sh-DSCR8 had the contrary effect on Hep3B cells' apoptosis (Fig. 2f). Thus we conclude that DSCR8 promotes proliferation and cell cycle, while induces apoptosis in HCC cells.

### DSCR8 promotes HCC growth in vivo

**Fig. 3:**
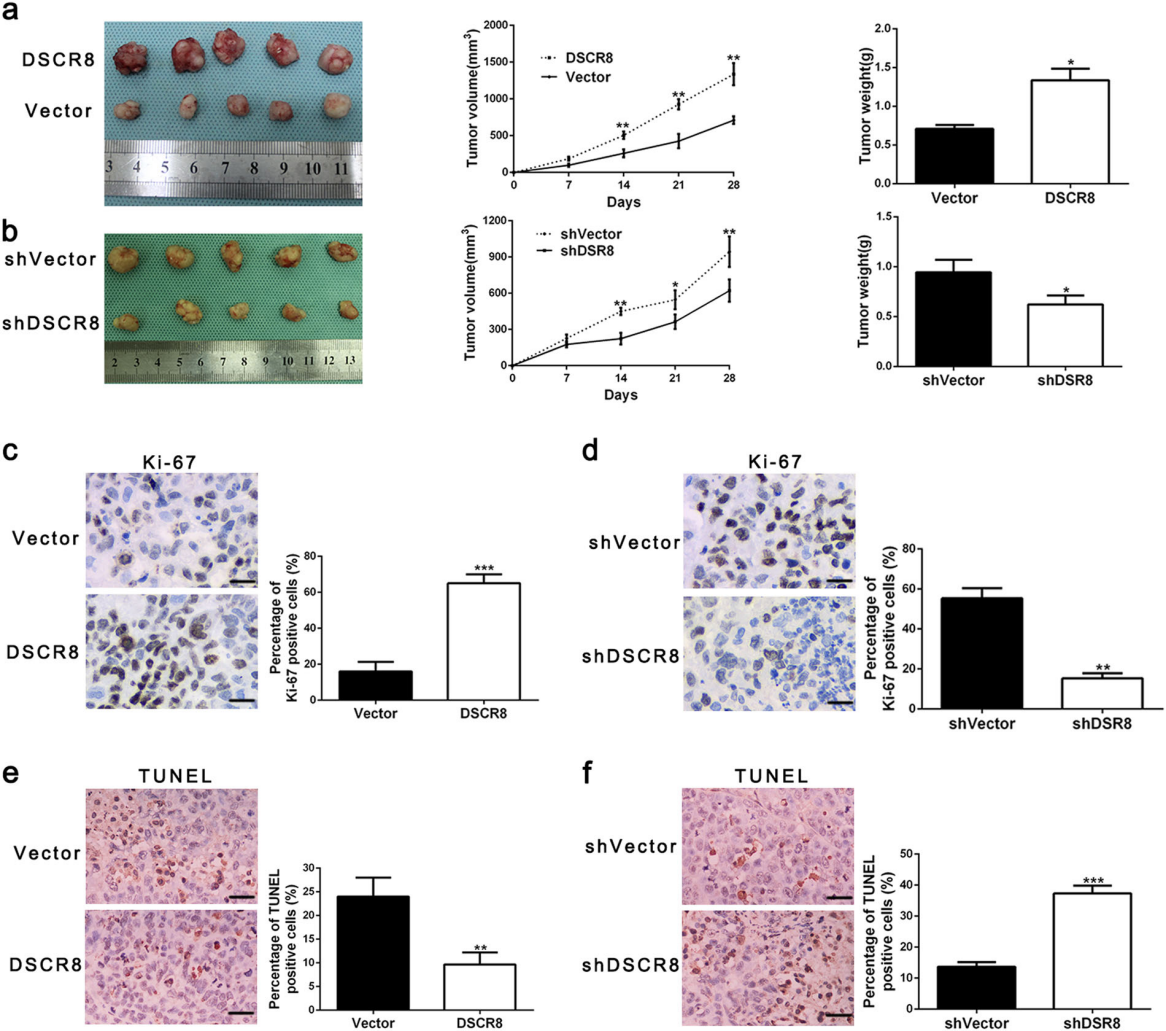
DSCR8 promotes HCC growth in vivo. **a** Tumor nodes with DSCR8 clones had larger tumor volume (middle panel) and heavier weight (right panel) than these with vectors. **b** Tumor nodes with shVector-Huh7 had larger tumor volume (middle panel) and heavier weight (right panel) than these with sh-DSCR8. Immunohistochemical staining in the xenografted tissues for Ki-67 (magnification: ×400. Bars: 50 µM.) in tumor nodule tissues showed that DSCR8 clone increased (**c**), whereas sh-DSCR8 decreased the percentage of Ki-67-positive cells (**d**). TUNEL assay in the xenografted tissues (magnification: ×400. Bars: 50 µM.) showed that DSCR8 clone reduced the proportion of apoptotic cells (**e**), whereas sh-DSCR8 increased the percentage (**f**). Bars: 50 µM. *P < 0.05, **P < 0.01, ***P < 0.001

Next, subcutaneous tumor models were conducted to explore the effects of DSCR8 in vivo. We found that the sizes and weights of tumor nodes were increased compared by pcDNA/DSCR8 compared to control group (Fig. 3a), while sh-DSCR8 resulted in decreases in the sizes and weights of tumor nodes (Fig. 3b). Immunohistochemistry for Ki-67 and terminal deoxinucleotidyl transferase-mediated dUTP-fluorescein nick end labeling (TUNEL) staining in the xenografted tissues revealed that pcDNA/DSCR8 increased the proportion of Ki-67-positive cells (Fig. 3c) and reduced the proportion of apoptotic cells (Fig. 3e), while sh-DSCR8 had the contrary effects (Fig. 3d, f). Thus these findings demonstrate that DSCR8 promotes HCC growth both in vitro and in vivo.

### DSCR8 acts as a molecular sponge for miR-485-5p in HCC cells

**Fig. 4:**
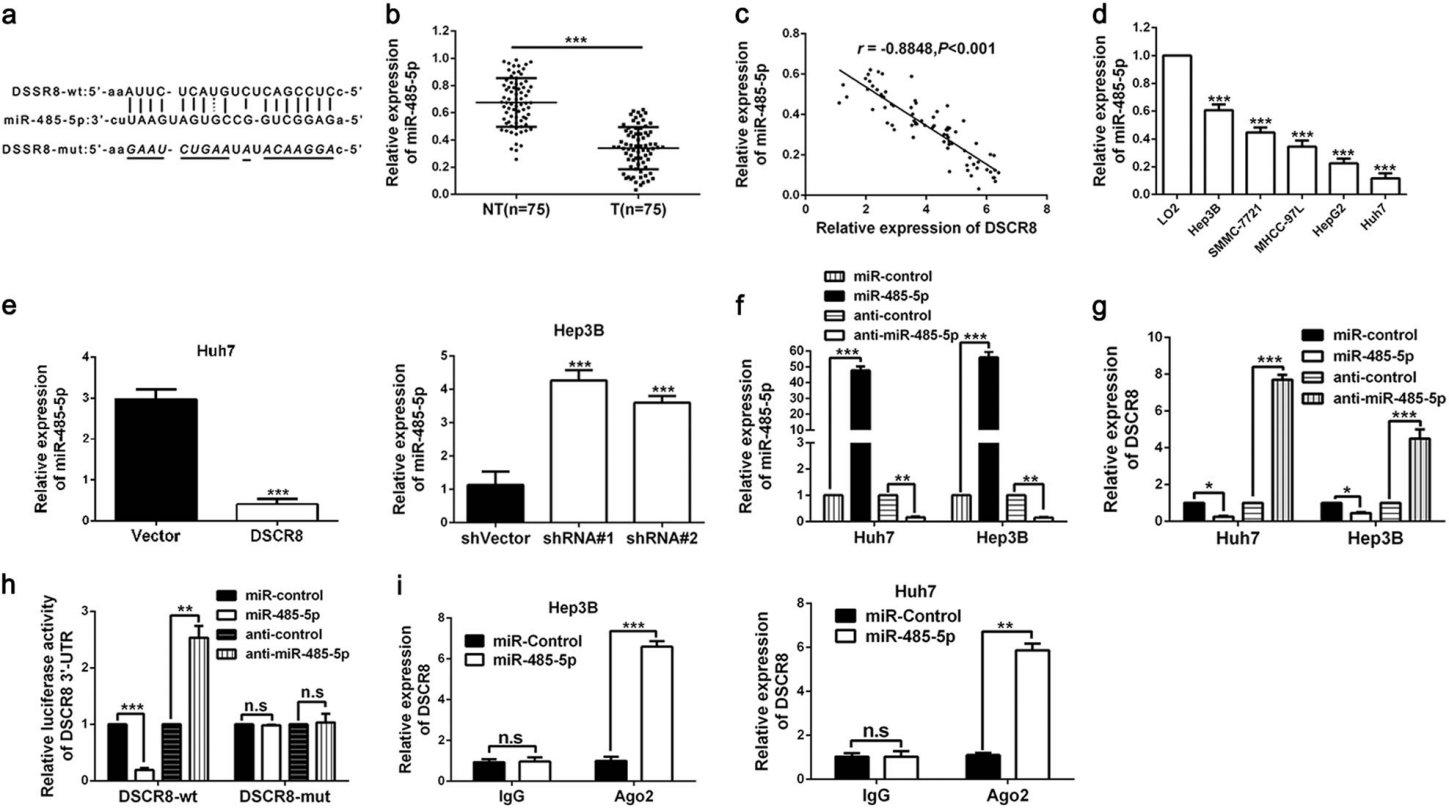
DSCR8 functions as a sponge for miR-485-5p. **a** By applying bioinformatics tools (microRNA.org, TargetScan, and miRDB), we found that there were putative binding sites between 3′UTR of DSCR8-wild type (wt) and miR-485-5p. DSCR8-mutant (mut) means mutation of binding sites in the 3′UTR of DSCR8. **b** The expression of miR-485-5p in tumor tissues (*n* = 75) was significantly lower than that in adjacent non-tumor tissues (*n* = 75). **c** Pearson correlation analysis revealed that there existed a negative association between miR-485-5p and DSCR8 in HCC tissues. **d** MiR-485-5p was downregulated in HCC cell lines. **e** Real-time PCR showed that miR-485-5p was negatively regulated by DSCR8. **f** MiR-485-5p expression was significantly increased by miR-485-5p mimics, whereas decreased by the inhibitors. **g** The expression of DSCR8 was negatively regulated by DSCR8. **h** Luciferase reporter gene assays showed that miR-485-5p negatively regulated the luciferase activity of DSCR8-wt-3′UTR, rather than of DSCR8-mut-3′UTR. **i** The anti-Ago2 RIP assay with miR-485-5p mimics showed that both miR-485-5p and DSCR8 were enriched in Ago2 precipitate compared to IgG. *n* *=* three independent experiments. *P < 0.05, **P < 0.01, ***P < 0.001

Previous evidences reveal that lncRNAs is capable of acting as a molecular sponge for miRNAs to participate in the cancer progression^19^. And to explore whether DSCR8 could act as a molecular sponge for miRNAs in HCC cells, RNA fluorescence in situ hybridization (RNA-FISH) for the subcellular localization of DSCR8 was conducted. The result indicated that DSCR8 was localized both in the cell nuclear and cytoplasm (Supplementary Fig. 1), which suggested that DSCR8 could act as a molecular sponge for miRNAs. Then we attempted to determine the most potential miRNA that can be regulated by DSCR8 via applying bioinformatics tools (microRNA.org, TargetScan, and miRDB). The data suggested that there were putative binding sites between DSCR8 and miR-485-5p (Fig. 4a). In addition, miR-485-5p has been identified as an anti-oncogene in HCC^11,12^. Thus we focused on miR-485-5p. By performing real-time PCR in all of the 75 paired HCC tissues and the adjacent non-tumor tissues, we confirmed that miR-485-5p was significantly underexpressed in HCC tissues (*P* < 0.001, Fig. 4b). Pearson correlation analysis indicated that DSCR8 expression was negatively related to miR-485-5p expression in HCC tissues (*r* = −0.8848, *P* < 0.001, Fig. 4c). In addition, the expression of miR-485-5p was underexpressed in HCC cell lines compared to LO2 cells (*P* < 0.001, respectively, Fig. 4d). Furthermore, miR-485-5p was negatively regulated by DSCR8 in Huh7-pcDNA/DSCR8 and Hep3B-sh-DSCR8 cells (Fig. 4e). We altered the miR-485-5p expression levels of Huh7 and Hep3B cells by miR-485-5p mimics and inhibitors (Fig. 4f). Interestingly, the expression of DSCR8 was negatively regulated by miR-485-5p (Fig. 4g). Furthermore, luciferase reporter gene assay revealed that miR-485-5p directly targeted 3′UTR of DSCR8-wt to negatively regulate the luciferase activity of DSCR8-wt-3′UTR, rather than 3′UTR of DSCR8-mut (Fig. 4h). Then anti-Ago2 RNA immunoprecipitation (RIP) assay was conducted with miR-485-5p mimics, and the data revealed that both DSCR8 and miR-485-5p were enriched in pulled down Ago2 protein (Fig. 4i). Taken together, the above findings indicate that miR-485-5p is a downstream target of DSCR8 in HCC cells.

### miR-485-5p mediates the effects of DSCR8 on proliferation, cell cycle, and apoptosis of HCC cells

**Fig. 5:**
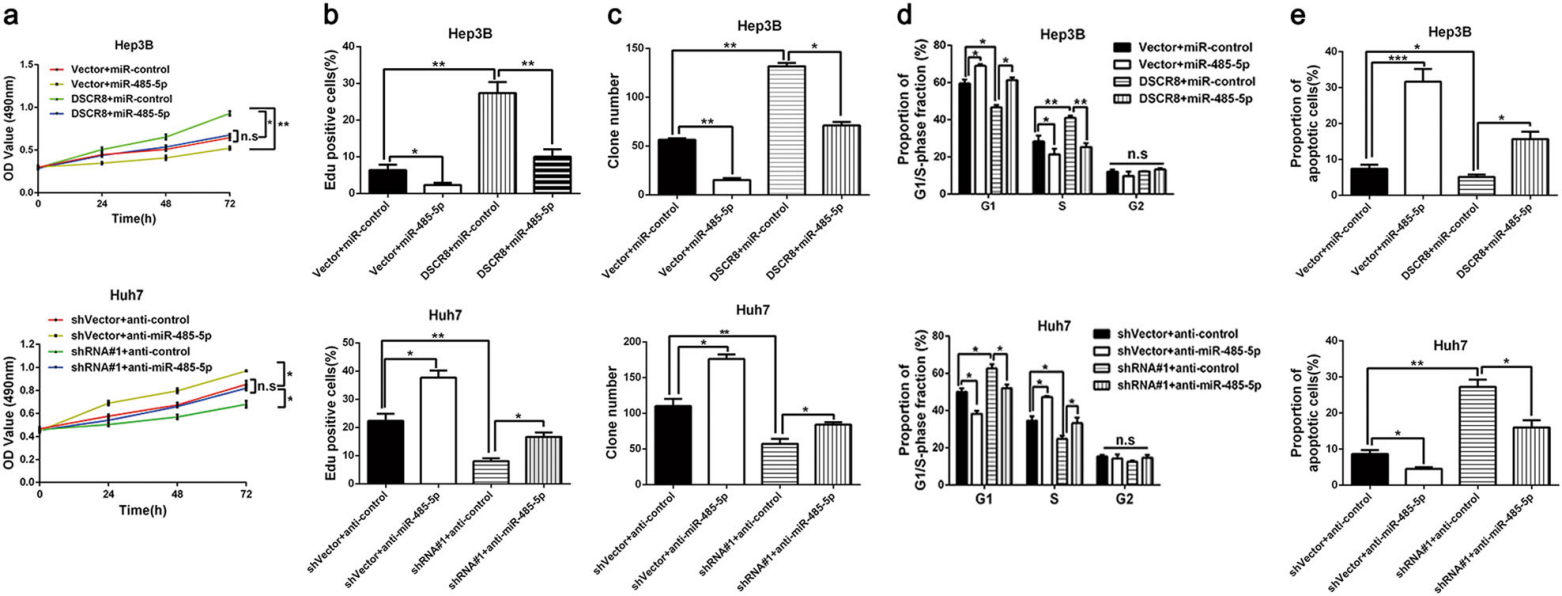
The functions of DSCR8 is mediated by miR-485-5p. miR-485-5p mimics suppressed, whereas miR-485-5p inhibitors promoted HCC cells' proliferation (**a**–**c**) and cell cycle (**d**). Meanwhile, miR-485-5p mimics induced, whereas miR-485-5p inhibitors inhibited HCC cells' apoptosis (**e**). miR-485-5p mimics partially offset the promotion effects of DSCR8 on Hep 3B cells' proliferation (**a**–**c**) and cell cycle (**d**) while reversed the inhibitory effects of DSCR8 on cells' apoptosis (**e**). miR-485-5p inhibitors reversed the inhibitory effects of sh-DSCR8 on Huh7 cells' proliferation (**a**–**c**) and cell cycle (**d**) while receded the promotion effects of sh-DSCR8 on cells' apoptosis (**e**). *n* *=* three independent experiments. *P < 0.05, **P < 0.01, ***P < 0.001

Next, we conducted rescue experiments to explore whether miR-485-5p mediated the effects of DSCR8 on cell proliferation, cell cycle, and cell apoptosis in HCC cells. Rescue experiments in Hep3B cells revealed that miR-485-5p mimics receded the promotion effect of pcDNA/DSCR8 on cell proliferation (Fig. 5a–c) and cell cycle (Fig. 5d) and reversed the inhibitory effect of DSCR8 on cell apoptosis (Fig. 5e). In contrast, miR-485-5p inhibitors reversed the inhibitory effect of sh-DSCR8 on cell proliferation (Fig. 5a–c) and cell cycle (Fig. 5d) in Huh7 cells while weakened the promotion effect on apoptosis (Fig. 5e). Thus we demonstrate that miR-485-5p mediates the effects of DSCR8 on proliferation, cell cycle, and apoptosis of HCC cells.

### MiR-485-5p inhibits Wnt/β-catenin signal pathway by directly targeting FZD7 in HCC cells

**Fig. 6:**
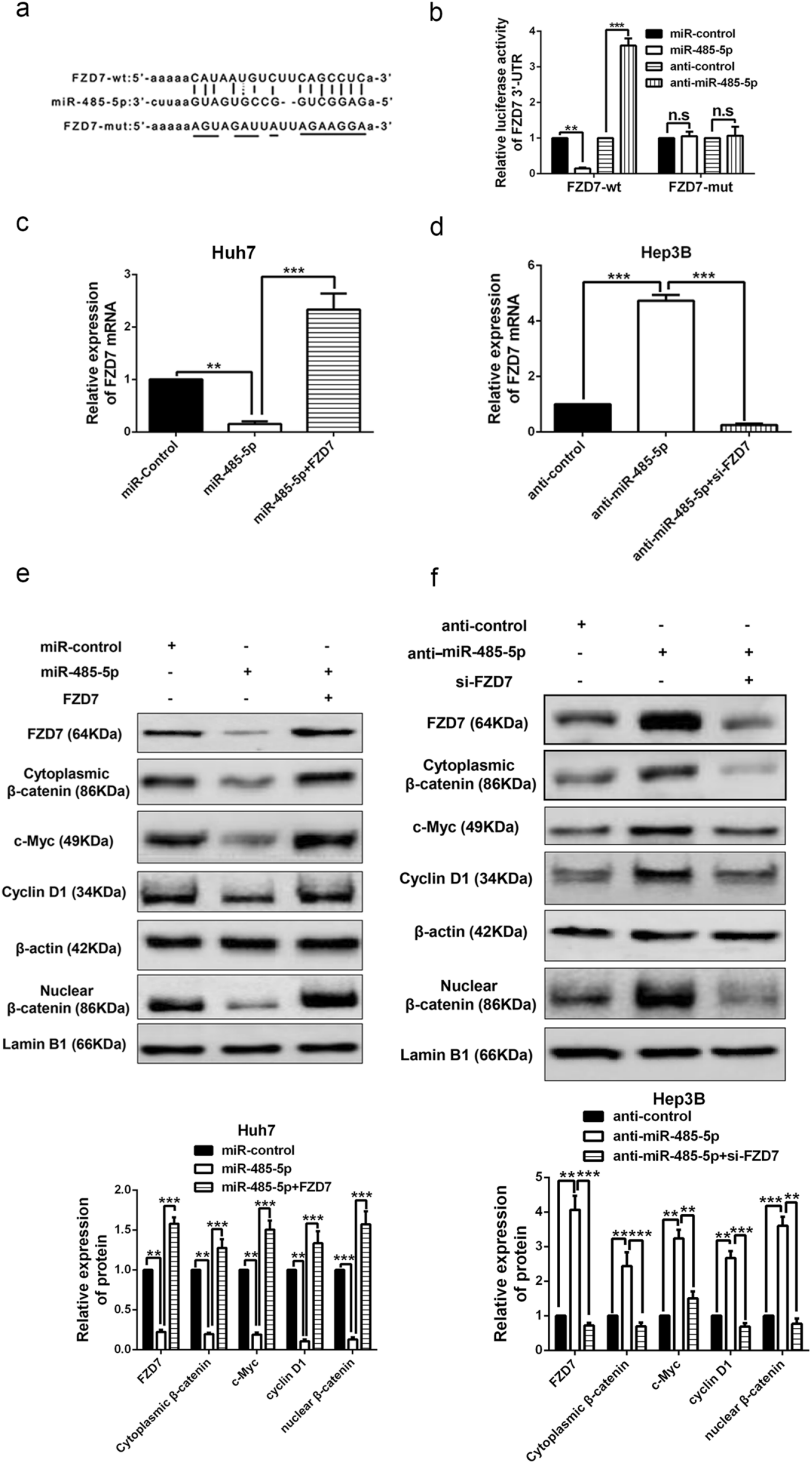
miR-485-5p inhibits Wnt/β-catenin pathway by directly targeting FZD7. **a** Data from bioinformatics tools (microRNA.org, TargetScan, and miRDB) showed that there were putative binding sites between 3′UTR of FZD7-wt and miR-485-5p. FZD7-mut means mutation of binding sites in 3′UTR of FZD7. **b** Luciferase reporter gene assays revealed that miR-485-5p negatively regulated the luciferase activity of FZD7-wt-3′UTR, rather than of DSCR8-mut-3′UTR. The mRNA (**c**, **d**) and protein (**e**, **f**) expression of FZD7 was negatively regulated by miR-485-5p. And FZD7 clone or siRNAs reversed the effects of miR-485-5p mimics or inhibitors on FZD7 expression (**c**–**f**). **e**, **f** Western blot results revealed that the accumulation of cytoplasmic β-catenin and the accumulation of nuclear β-catenin, c-Myc expression, and cyclin D1 expression were negatively regulated by miR-485-5p, while reversed by FZD7 clone and siRNA. *n* = three repeats with similar results, **P < 0.01, ***P *<* 0.001

Bioinformatics tools (microRNA.org, TargetScan, and miRDB) revealed that the 3′UTR of FZD7, which is a very critical receptor for activation of Wnt/β-catenin signal pathway^13,17^, had binding sites for miR-485-5p (Fig. 6a). In addition, it has been reported that FZD7 is a target of miR-485-5p in melanoma cells^18^. Then we attempted to explore whether FZD7 was a target of miR-485-5p in HCC cells. As showed in supplementary Fig. 2a, western blot results from our patients' cohort revealed that FZD7 was significantly upregulated in HCC tissues than that in normal tissues, which was consistent with the data from database UALCAN (http://ualcan.path.uab.edu/index.html; supplementary Fig. 2b). Luciferase reporter gene indicated that miR-485-5p negatively regulated the fluorescence intensity of FZD7-wt-3′UTR, rather than FZD7-mut-3′UTR (Fig. 6b). Real-time PCR revealed that FZD7 mRNA (Fig. 6c, d) and protein expression levels (Fig. 6e, f) of Hep3B and Huh7 cells were negatively regulated by miR-485-5p. In addition, we found that miR-485-5p mimics also decreased the accumulation of both cytoplasmic and nuclear β-catenin, and the expression of Wnt/β-catenin pathway downstream targets c-Myc and cyclin D1 in Huh7 cells (Fig. 6e). On the other hand, miR-485-5p inhibitors had the contrary effects in Hep3B cells (Fig. 6f). In contrast, FZD7 reversed the effects of miR-485-5p on these proteins’ expression (Fig. 6c–f). Thus we conclude that miR-485-5p directly targets FZD7 to inhibit Wnt/β-catenin signal pathway in HCC cells.

### DSCR8 activates Wnt/β-catenin signal pathway by DSCR8/miR-485-5p/FZD7 axis

**Fig. 7:**
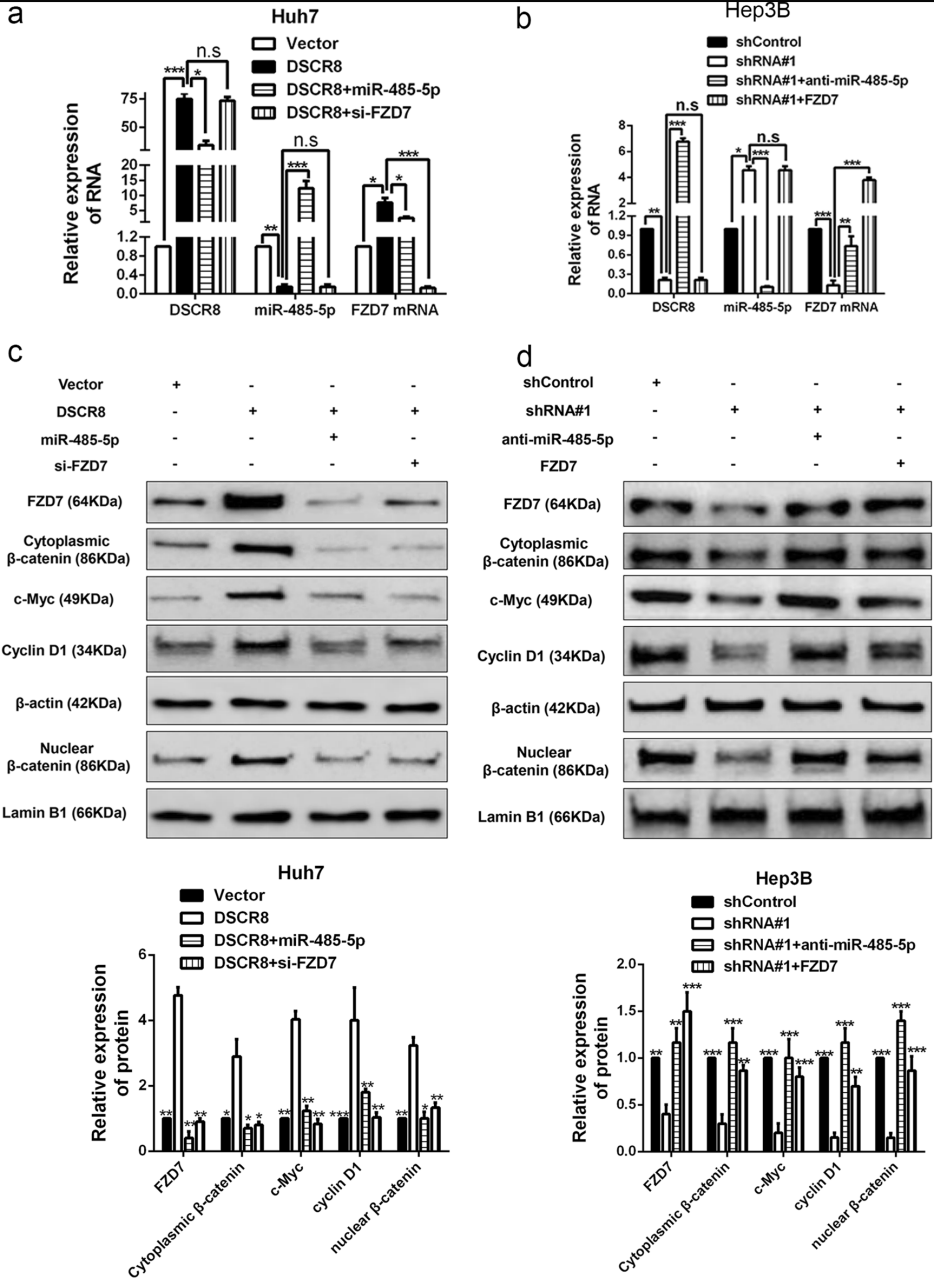
DSCR8 activates Wnt/β-catenin pathway by DSCR8/miR-485-5p/FZD7 axis. Rescue experiments revealed that DSCR8 negatively regulated the miR-485-5p expression (**a**, **b**), whereas positively regulated both the mRNA (**a**, **b**) and protein (**c**, **d**) expression of FZD7 by DSCR8/miR-485-5p/FZD7 axis. **c**, **d** Western blot results from rescue experiments showed that DSCR8 positively regulated the accumulation of cytoplasmic β-catenin and the accumulation of nuclear β-catenin, c-Myc expression, and cyclin D1 expression by DSCR8/miR-485-5p/FZD7 axis. *n* = three repeats with similar results, *P < 0.05, **P < 0.01, ***P *<* 0.001

Then we attempted to explore whether DSCR8 activated Wnt/β-catenin signal pathway by DSCR8/miR-485-5p/FZD7 axis. Real-time PCR for rescue experiments revealed that DSCR8 positively regulated FZD7 mRNA expression, which was mediated by miR-485-5p (Fig. 7a, b). Western blot in Hep3B cells revealed that pcDNA/DSCR8 not only increased the expression of FZD7 but also increased the accumulation of both cytoplasmic and nuclear β-catenin and the expression of c-Myc and cyclin D1, while miR-485-5p mimics or FZD7 clone reversed the effects of pcDNA/DSCR8 (Fig. 7c). On the contrary, sh-DSCR8 decreased the expression of FZD7, the accumulation of both cytoplasmic and nuclear β-catenin, and the expression of c-Myc and cyclin D1, while miR-485-5p inhibitors or FZD7 small interfering RNA (siRNA) reversed the effects of sh-DSCR8 (Fig. 7a, d). Taken together, the above data demonstrate that DSCR8 activates Wnt/β-catenin signal pathway by DSCR8/miR-485-5p/FZD7 axis.

### The clinical significances of DSCR8 and miR-485-5p in HCC

**Fig. 8:**
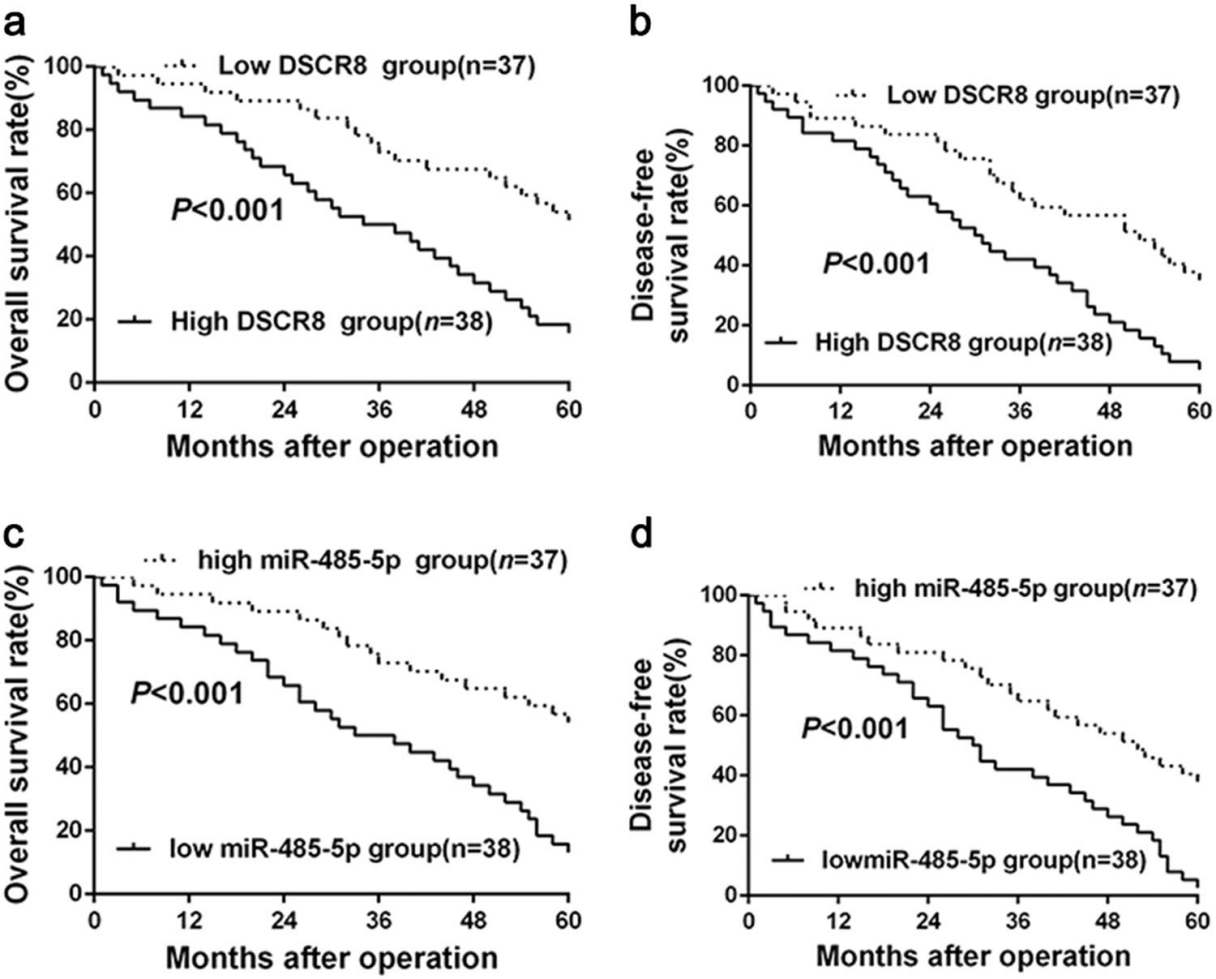
The prognostic value of DSCR8 and miR-485-5p expression in HCC patients. HCC patients in high DSCR8 group had poorer 5-year overall survival (**a**) and disease-free survival (**b**). MiR-485-5p high-expressing HCC patients showed better 5-year overall survival (**c**) and disease-free survival (**d**) compared to miR-485-5p low-expressing cases. For each cohort, different subgroups were sorted according to the median expression levels of DSCR8 and miR-485-5p

Next, based on the median expression levels of DSCR8 and miR-485-5p in HCC tissues, 75 HCC tissues were, respectively, sorted into two subgroups (high/low DSCR8 group, high/low miR-485-5p group). Statistical analysis revealed that DSCR8 was closely related to tumor size (*P* = 0.015) and tumor–node–metastasis (TNM) stage (*P* = 0.020) (Table 1). And miR-485-5p was closely related to tumor size (*P* = 0.027), venous infiltration (*P* = 0.025), and Edmondson–Steiner grading (*P* = 0.019) (Table 2). In addition, patients with high DSCR8 had poorer 5-year overall survival (*P* < 0.001, Fig. 8a) and disease-free survival (*P* < 0.001, Fig. 8b). In contrast, patients with low miR-485-5p had worse 5-year overall survival (*P* < 0.001, Fig. 8c) and disease-free survival (*P* < 0.001, Fig. 8d). Taken together, these data indicate that DSCR8 and miR-485-5p may be novel indicators for diagnosis and prognosis of HCC.

**Table 1:** Correlation between DSCR8 expression and the clinicopathologic characteristics in HCC

Characteristics		Cases (*n* = 75)	Number of patients		*P*
			DSCR8^high^ (*n* = 38)	DSCR8 ^low^ (*n* = 37)	
Age (years)	<50	24	10	14	0.285
	≥50	51	28	23	
Gender	Male	64	33	31	0.708
	Female	11	5	6	
HBV	Absent	13	7	6	0.801
	Present	62	31	31	
Serum AFP level (ng/mL)	<400	18	8	10	0.545
	≥400	57	30	27	
Tumor size (cm)	<5	34	12	22	**0.015**
	≥5	41	26	15	
Number of tumor nodules	1	62	32	30	0.720
	≥2	13	6	7	
Cirrhosis	Absent	20	12	8	0.330
	Present	55	26	29	
Venous infiltration	Absent	54	24	30	0.084
	Present	21	14	7	
Edmondson–Steiner grading	I+II	49	22	27	0.170
	III+IV	26	16	10	
TNM stage	I+II	56	24	32	**0.020**
	III+IV	19	14	5	

*HCC* hepatocellular carcinoma, *HBV* hepatitis B virus, *AFP* alpha-fetoprotein, *TNM* tumor–node–metastasis

The bold values means their *P*-values <0.05

**Table 2:** Correlation between miR-485-5p expression and the clinicopathologic characteristics in HCC

Characteristics		Cases (*n* = 75)	Number of patients		*P*
			miR-485-5p^low^ (*n* = 38)	miR-485-5p^high^ (*n* = 37)	
Age (years)	<50	24	11	13	0.566
	≥50	51	27	24	
Gender	Male	64	33	31	0.708
	Female	11	5	6	
HBV	Absent	13	8	5	0.389
	Present	62	30	32	
Serum AFP level (ng/mL)	<400	18	8	10	0.545
	≥400	57	30	27	
Tumor size (cm)	<5	34	22	12	**0.027**
	≥5	41	16	25	
Number of tumor nodules	1	62	30	32	0.389
	≥2	13	8	5	
Cirrhosis	Absent	20	7	13	0.102
	Present	55	31	24	
Venous infiltration	Absent	54	23	31	**0.025**
	Present	21	15	6	
Edmondson–Steiner grading	I+II	49	20	29	**0.019**
	III+IV	26	18	8	
TNM stage	I+II	56	26	30	0.208
	III+IV	19	12	7	

*HCC* hepatocellular carcinoma, *HBV* hepatitis B virus, *AFP* alpha-fetoprotein, *TNM* tumor–node–metastasis

The bold values means their *P*-values <0.05.

## Discussion

Growing evidences confirm that non-coding RNAs, especially lncRNAs and miRNAs, have considerable potential value for improving diagnostic and therapeutic levels for HCC^20,21^. Recently, researchers have identified numerous lncRNAs and miRNAs in HCC, such as lncRNA HNF1A-AS1^22^, LncRNA-NEF^22^, miRNA-874^23^, and so on. In these researches, abnormally expressed non-coding RNAs involve in various cytological behaviors of HCC cells^22,23^, which suggests that lncRNAs and miRNAs deserve further studies. LncRNA DSCR8 is a newly identified non-coding RNA in uterine cancer and melanoma, which might be potential prognostic indicators and therapeutic targets in these cancers^9,10^. However, the expression and functions of DSCR8 in HCC remain uncovered. In the present study, we reported the expression, functions, and potential mechanism of DSCR8 in HCC for the first time. Our study revealed that DSCR8 was overexpressed in HCC tissues and cell lines, which was consistent with the data from GEO dataset. These findings suggests that DSCR8 may be a potential oncogene in HCC.

Then we explored the functions of DSCR8 on cell proliferation, cell cycle, and cell apoptosis in HCC by gain- and loss-of-function experiments. As we expected, upregulated DSCR8 promoted cell proliferation and cell cycle and repressed cell apoptosis, whereas silenced DSCR8 had the contrary effects. Besides, we confirmed the functions of DSCR8 in HCC by establishing nude mice models. These findings demonstrates that DSCR8 plays important roles in HCC growth.

Next, we explored the potential mechanism of DSCR8 in HCC cells. It is well known that lncRNAs exert their influences through multiple ways among which acting as ceRNAs is a very general and an important path for lncRNAs to exert the influences. First, we conducted RNA-FISH assay to explore the subcellular location of DSCR8 in HCC cells. The result indicated that DSCR8 was localized both in the cell nuclear and cytoplasm, which suggested that DSCR8 could act as a molecular sponge for miRNAs. Then, based on the analysis results from bioinformatics tools and previous studies^11,12^, we speculated that DSCR8 might act as the sponge for miR-485-5p in HCC cells. Interestingly, we found that miR-485-5p was significantly underexpressed in HCC tissues and cell lines. And there existed a negative correlation between DSCR8 expression and miR-485-5p expression in HCC. In addition, the expression of miR-485-5p was negatively regulated by DSCR8 in HCC cells. Meanwhile, miR-485-5p also negatively regulated DSCR8. Subsequently, luciferase reporter gene and anti-Ago2 RIP revealed that DSCR8 directly targeted miR-485-5p in HCC cells. What is more, rescue experiments in in vitro experiments manifested that miR-485-5p was a mediator for DSCR8 in HCC cells. These data strongly demonstrate that DSCR8 may act as a sponge for miR-485-5p in HCC cells.

Furthermore, we attempted to explore the downstream target of miR-485-5p in HCC cells, which may mediate DSCR8/miR-485-5p axis. Then bioinformatics tools combined with previous studies^18^ were employed for comprehensive analysis. Based on the bioinformatics tools, we found that FZD7, a very critical receptor for activation of Wnt/β-catenin signal pathway^13^, may be one of the targets of miR-485-5p. Besides, it has been reported that FZD7 is a target of miR-485-5p in melanoma cells^18^, and FZD7 is an oncogene in HCC^24^. Results from our patients' cohort confirmed that FZD7 was significantly upregulated in HCC tissues than that in normal tissues, which was consistent with the data from database UALCAN (http://ualcan.path.uab.edu/index.html). Then luciferase reporter gene indicated that miR-485-5p targeted FZD7. In addition, real-time PCR and western blot for rescue experiments showed that miR-485-5p negatively regulated the expression of FZD7, the accumulation of both cytoplasmic and nuclear β-catenin, and the expression of Wnt/β-catenin pathway downstream targets c-Myc and cyclin D1 in HCC cells. The above data revealed that miR-485-5p directly targeted FZD7 to inhibit Wnt/β-catenin signal pathway in HCC cells. Our above findings revealed that miR-485-5p was a mediator for DSCR8 in HCC cells; then we attempted to explore the relationships among DSCR8, miR-485-5p, FZD7, and Wnt/β-catenin pathway. In the subsequent rescue experiments, we measured the expression changes of FZD7, the accumulation of both cytoplasmic and nuclear β-catenin, and the expression of c-Myc and cyclin D1. We found that miR-485-5p or FZD7 mediated DSCR8-induced activation of Wnt/β-catenin pathway. Thus we conclude that DSCR8 activates Wnt/β-catenin signal pathway to promote HCC progression by DSCR8/miR-485-5p/FZD7 axis. Clinically, we found that DSCR8 and miR-485-5p were closely related to tumor size, TNM stage, venous invasion, 5-year overall survival, and 5-year disease-free survival of HCC patients.

**Fig. 9:**
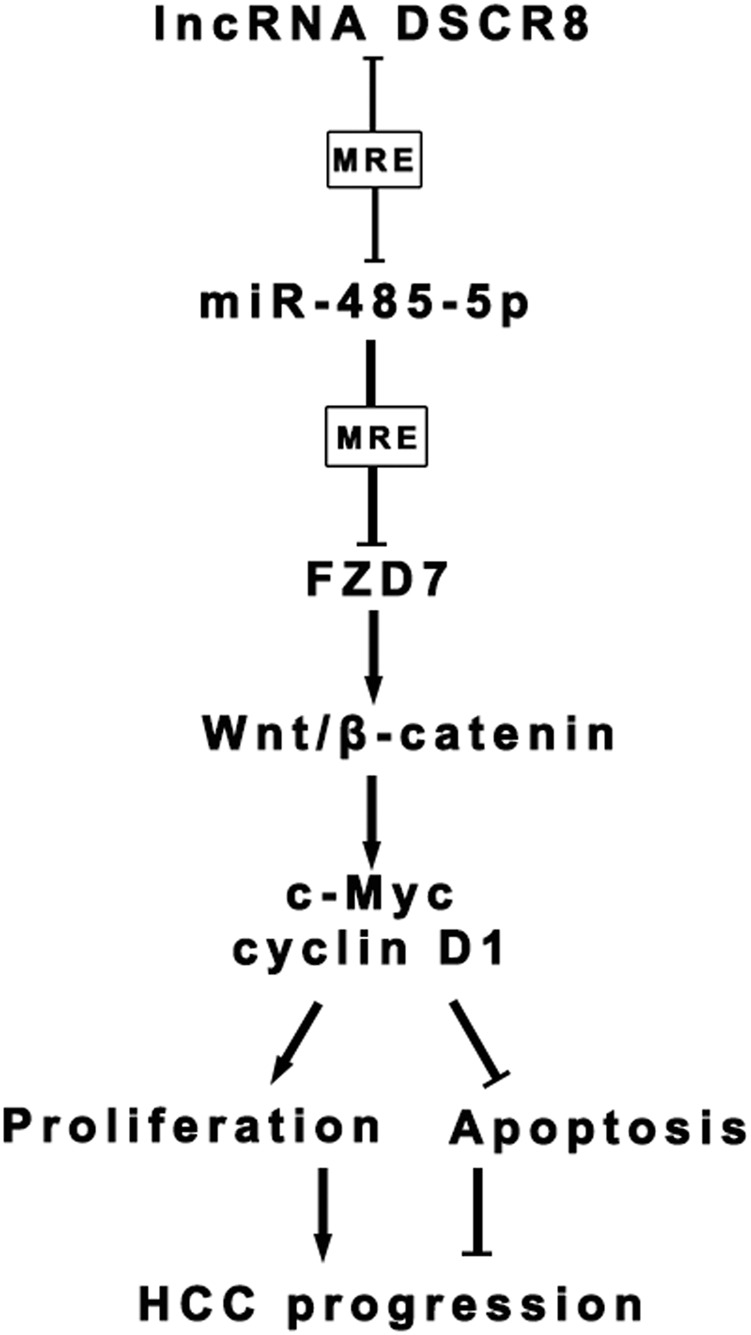
Schematic of the proposed mechanism of DSCR8 in HCC. DSCR8 acts as a molecular sponge for miR-485-5p to regulate FZD7 expression, subsequently activating Wnt/β-catenin signal pathway to promote HCC progression

Herein, the present study determines the expression, clinical significance, and functions of lncRNA DSCR8 in HCC for the first time. In addition, we found that DSCR8 activates Wnt/β-catenin signal pathway to promote HCC progression by DSCR8/miR-485-5p/FZD7 axis (Fig. 9). Our study provides a novel potential targeted therapy strategy for HCC.

## Materials and methods

### Tissue samples

HCC tissue samples and adjacent non-tumor tissue samples, which were histopathologically confirmed, were collected from 75 HCC patients who underwent surgery at the First Affiliated Hospital of Xi’an Jiaotong University from January 2009 to December 2011. All of the patients did not receive chemotherapy or radiotherapy before surgery. All of the samples were stored at −80 °C. Our study got approval from the Ethics Committees of the First Affiliated Hospital of Xi’an Jiaotong University, and informed consent was obtained from all of the patients.

### Cell culture

The human normal liver cell (LO2) and HCC cell lines (MHCC-97 L, HepG2, Hep3B, Huh7, and SMMC-7721) were purchased from the Cell Bank of the Chinese Academy of Sciences (Shanghai, China). All of the cells were maintained in incubator (37 °C, 5% CO_2_) and cultured in Dulbecco’s modified Eagle’s medium (Gibco, Grand Island, NY, USA) supplemented with 10% fetal bovine serum (FBS; Gibco, Grand Island, NY, USA) and 1% penicillin–streptomycin (Invitrogen, CA, USA).

### RNA fluorescent in situ hybridization

Subcellular localization of DSCR8 was detected by the FISH Kit (RiboBio, Guangzhou, China) according to the manufacturer’s instructions. Cy3-labeled DSCR8 probe were obtained from RiboBio (Guangzhou, China). In brief, HCC cells (2 × 10^4^) were seeded on cell slides in 24-well culture plates. After fishing induction, cells were fixed in 4% paraformaldehyde for 30 min at room temperature. After permeabilization, cells were prehybridized with prehybridization solution and hybridization solution, then incubated with the cy3-labeled DSCR8 oligonucleotide probe. Cell nuclei were stained with 4,6-diamidino-2-phenylindole for 5 min at room temperature. Fluorescence images were obtained by laser scanning confocal microscope (LSM700, Carl Zeiss, Germany).

### Cell transfection

The assays were performed as described previously^6,7^. Scrambled shRNA (shControl) and DSCR8 shRNAs (shRNA#1 and shRNA#2), as well as pcDNA3.1-Control (Vector) and pcDNA3.1-DSCR8 (DSCR8) were purchased from Invitrogen (Carlsbad, CA, USA). MiR-485-5p inhibitors (#HmiR-AN0520-AM02) and miR-485-5p mimics (#HmiR0239-MR04) were purchased from Genecopoeia (Guangzhou, China). FZD7 Human cDNA ORF Clone (FZD7) and FZD7 siRNA (si-FZD7) were purchased from OriGene (OriGene Technologies,Inc., USA).

### Real-time PCR

The assay was conducted according to the protocols described in our previous studies^7^. All RNA was extracted based on the protocol of TRIzol reagent (Invitrogen, Carlsbad, CA, USA). In brief, for detection of miRNA expression, cDNA was synthesized using the qScript microRNA cDNA Synthesis Kit (Quantabio, Beverly, MA) according to the manufacturer’s instruction. Quantitative PCR was performed with the miScript SYBR Green PCR Kit (Qiagen, Hilden, Germany) under the following thermocycler conditions: 95 °C for 2 min, and 40 cycles of 95 °C for 5 s and 60 °C for 30 s. For detection of mRNA expression, the cDNAs were synthesized by the cDNA Synthesis Kit (Thermo Fisher Scientific, Waltham, MA, USA) under the following thermocycler conditions: 95 °C for 5 min, and 40 cycles of 95 °C 20 s and 62 °C 30 s, followed by 72 °C 3 min. Primers for DSCR8 (#HQP020874), miR-485-5p (#HmiRQP0520), snRNA U6 (#HmiRQP0520), FZD7 (#HQP020136), and GAPDH (#C0288) were ordered from Genecopoeia (Guangzhou, China).

### Detection of cell proliferation, cell cycle, and cell apoptosis

MTT assay, plate clone formation assay, Edu assay, flow cytometry for cell cycle, and cell apoptosis were conducted according to the protocols described in our previous studies^25,26^. Briefly, with respect to MTT assay, proliferation of cells was measured by MTT solution (5 mg/ml; Beyotime Institute of Biotechnology, Shanghai, China) at 24, 48, and 72 h, respectively. With respect to plate clone formation assay, 1 × 10^3^ cells were seeded in 6-well plates. The cells were mixed and then cultured for 2 weeks in culture medium with 10% FBS. Clusters containing ≥30 cells were counted as a single colony. With respect to Edu assay, Cell-Light™ EdU Apollo®488 In Vitro Imaging Kit (#C10310-3, RiboBio Co., LTD, Guangzhou, China) was used. With respect to flow cytometry for cell cycle and cell apoptosis, PE Annexin V Apoptosis Detection Kit I (#559763, Becton Dickinson bioscience, San Jose, CA, USA) and PI/RNase Staining Buffer (#550825, Becton Dickinson bioscience, San Jose, CA, USA) were used. All of the above experiments were performed according to the manufacturer’s protocols.

### Western blot

The assay was conducted according to the protocols described in our previous studies^6,25^. Primary antibodies include: FZD7 (1:1000, ab64636, Abcam, MA, USA), β-catenin (1:1000, ab32572, Abcam), Cyclin D1 (1:1000, ab134175, Abcam), c-Myc (1:1000, ab32072, Abcam), and β-actin (1:1000, ab8226, Abcam). And Lamin B1 (1:5000, ab194109, Abcam) served as a nuclear internal control.

### Immunohistochemistry

The assays were conducted according to the protocols described in our previous studies^25^. Ki-67 primary antibody (1:200, 9027S, Cell Signaling, USA) and the biotinylated secondary antibody (ZSGB-BIO, Beijing, China) were applied. In Situ Cell Death Detection Kit (Roche Group, Basel, Switzerland) was applied for TUNEL staining.

### Luciferase reporter assay and RIP assay

The assays were conducted according to the protocols described in our previous studies^6,7^. In brief, for luciferase reporter assay, the 3′UTR of DSCR8 or FZD7 was amplified by PCR and inserted downstream of the firefly luciferase reporter gene in the pEZX‐MT06 vector (Genecopoeia, Guangzhou, China). Point mutations of the miR‐485-5p targeting sites in the DSCR8 or FZD7 3′UTR were generated using the QuickChange Multiple Site‐directed Mutagenesis Kit (Stratagene, La Jolla, CA). Vectors were transfected using Lipofectamine 2000 (Invitrogen, Carlsbad, CA, USA) into HCC cells. Cells were collected 48 h later and the luciferase activity was quantified using the Luc-Pair™ Duo-Luciferase Assay Kit (Genecopoeia, Guangzhou, China). For RIP assay first, cells were collected and lysed in complete RIP lysis buffer. Then the cell extract was incubated with RIP buffer containing magnetic beads conjugated to a human anti-Ago2 antibody (Millipore, USA). Samples were incubated with proteinase K with shaking to digest proteins and the immunoprecipitated RNA was isolated. Subsequently, the NanoDrop spectrophotometer was used to measure the concentration of RNA, and the purified RNA was subjected to real-time PCR analysis.

### In vivo experiments

The assays were conducted according to the protocols described in our previous studies^25,26^. Twenty male BALB/c nude mice (4-week-old) were sorted into 4 groups with 5 mice in each group. Among them, two groups were subcutaneously injected into the flank of mice with 1 × 10^7^ Lv-DSCR8-Hep3B cells or 1 × 10^7^ Lv-NC-Hep3B cells, and the other two groups were conducted with 1 × 10^7^ Lv-anti-DSCR8-Huh7 cells or 1 × 10^7^ Lv-NC-Huh7 cells.

### Statistical analysis

SPSS 22.0 software (SPSS, Inc., Chicago, IL, USA) and Graphpad Prism 6.0 (San Diego, CA, USA) were applied to analyze the data. All of the data are presented as mean ± S.D. Statistical methods in this study included Student’s *t* test, one-way analysis of variance, Chi-square test, Kaplan–Meier method, log-rank test, Pearson’s correlation coefficient analysis, and so on. Difference with *P*<0.05 was considered to be statistically significant.

## Electronic supplementary material

Supplemental Figure 1

Supplemental Figure 2

Supplementary figure legends
