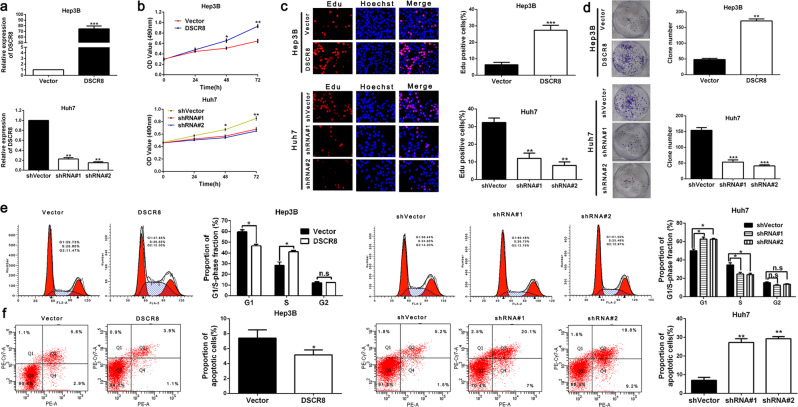# Correction: Long non-coding RNA DSCR8 acts as a molecular sponge for miR-485-5p to activate Wnt/β-catenin signal pathway in hepatocellular carcinoma

**DOI:** 10.1038/s41419-022-05141-9

**Published:** 2022-08-05

**Authors:** Yufeng Wang, Liankang Sun, Liang Wang, Zhikui Liu, Qing Li, Bowen Yao, Cong Wang, Tianxiang Chen, Kangsheng Tu, Qingguang Liu

**Affiliations:** Department of Hepatobiliary Surgery, The First Affiliated Hospital of Xi’an Jiaotong University, 710061 Xi’an, China

Correction to: *Cell Death and Disease* 10.1038/s41419-018-0937-7, published online 28 August 2018

The original version of this article unfortunately contained an error in a figure. In Figure 2C, the EdU staining of Hep3B cells in the control group was overexposed. To make the results clearer, the authors corrected the overexposed images. The authors confirm that this correction does not affect either the results or the conclusions of the paper. The corrected figure can be found below.