# Supplementary figures and images for: Long non-coding RNA DSCR8 acts as a molecular sponge for miR-485-5p to activate Wnt/β-catenin signal pathway in hepatocellular carcinoma
Source: Cell Death Dis. 2018 Aug 28;9(9):851. doi: 10.1038/s41419-018-0937-7 (PMC6113322; doi:10.1038/s41419-018-0937-7)

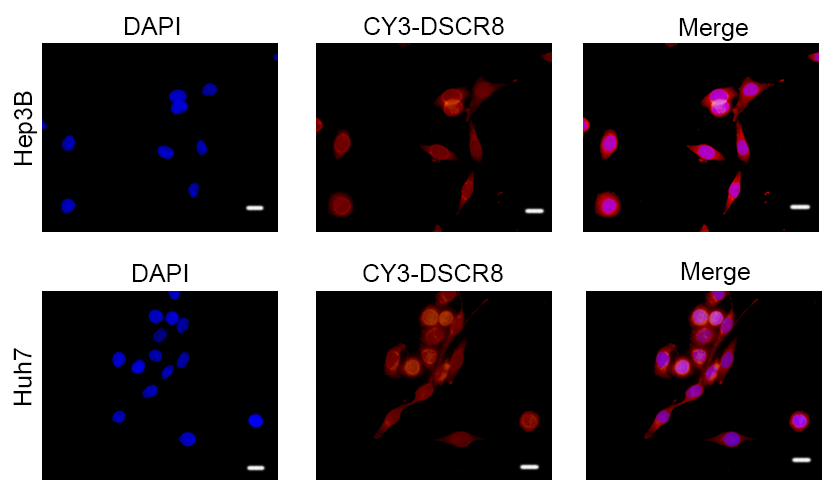

Supplement: Supplementary file 1 — Supplemental Figure 1 [file 41419_2018_937_MOESM1_ESM.tif]

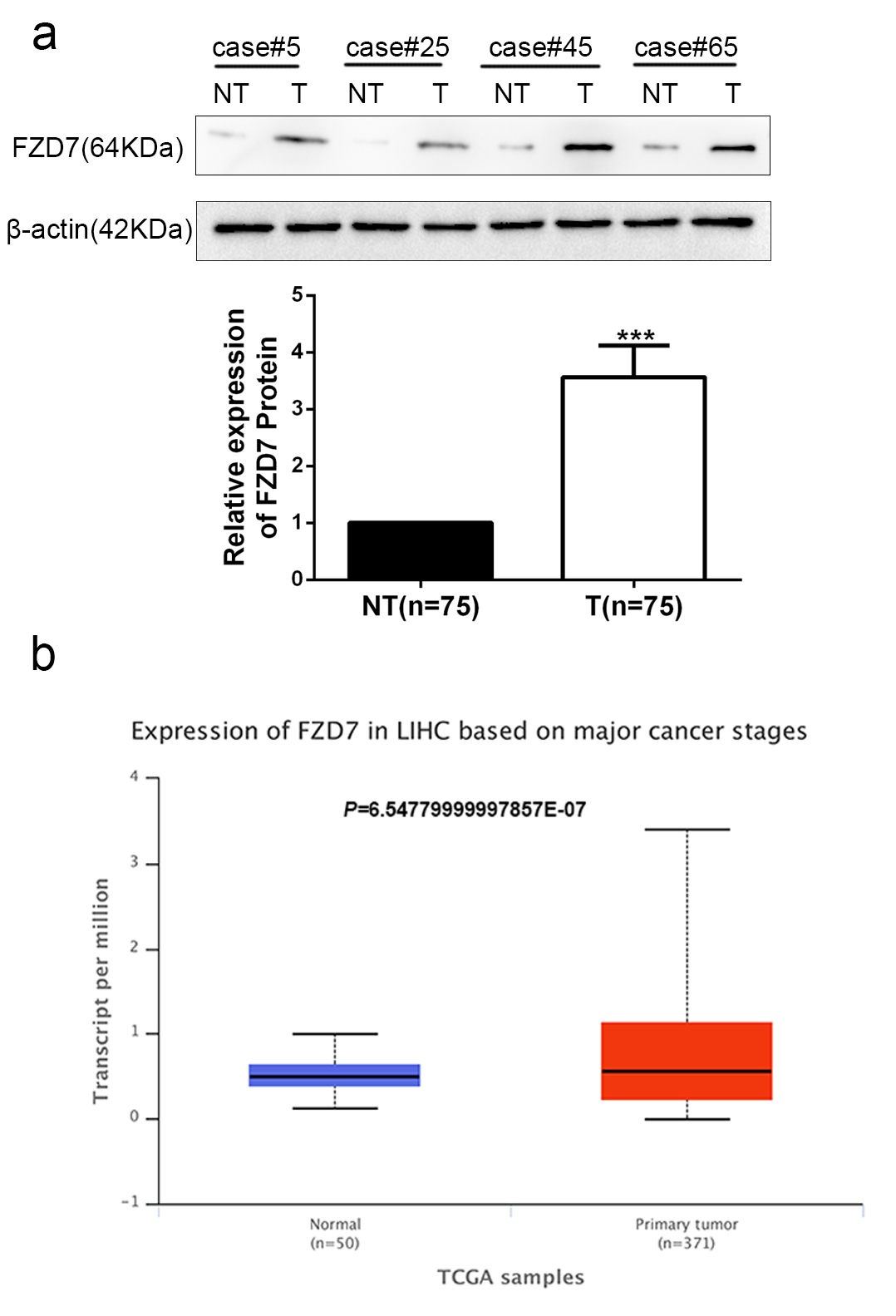

Supplement: Supplementary file 2 — Supplemental Figure 2 [file 41419_2018_937_MOESM2_ESM.tif]
