# Supplementary material for: Long non-coding RNA DSCR8 acts as a molecular sponge for miR-485-5p to activate Wnt/β-catenin signal pathway in hepatocellular carcinoma
Source: Cell Death Dis. 2018 Aug 28;9(9):851. doi: 10.1038/s41419-018-0937-7 (PMC6113322; doi:10.1038/s41419-018-0937-7)
Supplement: Supplementary file 3 — Supplementary figure legends [file 41419_2018_937_MOESM3_ESM.docx]

**Supplementary Fig. 1** Localization of DSCR8 is detected by RNA-FISH in HCC cells. Cy3 probes for DSCR8 staining, DAPI for nuclear staining. The result indicated that DSCR8 was localized both in the cell nuclear and cytoplasm. Bars: 10 µM.

**Supplementary Fig. 2** FZD7 is increased in HCC tissues. **a** Western blot results from our patients cohort revealed that DSCR8 was significantly up-regulated in HCC tissues (T, *n*=75) than that in normal tissues(NT, *n*=75). **b** Data from database UALCAN indicated that DSCR8 was significantly up-regulated in HCC tissues. ****P* < 0.001
